# How Much Importance Do We Give to Target Audiences in Article Writing?

**Published:** 2010

**Authors:** Sima Nedjat, Saharnaz Nedjat, Jaleh Gholami, Mahnaz Ashoorkhani, Katayoun Maleki, Soroush Mortaz Hejrie, Reza Majdzadeh

**Affiliations:** 1 Knowledge Utilization Research Centre (KURC), Tehran University of Medical Sciences, Tehran, Iran; 2 School of Public Health, Tehran University of Medical Sciences, Tehran, Iran

**Keywords:** Knowledge transfer, Research, Utilization, Iran, Audience, Message

## Abstract

**Objectives:**

Writing papers can be used as a means to convey a message. Knowledge transfer is also about conveying the right message to the right target audience. The aim of this study was to determine the proportion of articles that had mentioned a clear message and the target audience in the abstract and the article as a whole, and also to examine their association with different determinant factors.

**Methods:**

Articles published from 2001 to 2006 that were based on clinical and health system research conducted on Iranian populations and on maternal care, diabetes and tuberculosis were searched systematically in domestic and international databases. Eventually checklists (Additional file 1) were completed for 795 articles.

**Results:**

Overall, 98.5% of articles had a clear message, whereas 12.5% had addressed the direct target audience. Presence of a clear message in formatted abstracts were seen 3.6 times more (CI95%: 1.5-8.7) than in articles without formatted abstracts (p = 0.005). Addressing of the direct target audience was seen twice as much in health system research articles as compared to clinical studies, odds ratio was 2.3 (CI95%: 1.47-3.48, p<0.001).

**Conclusions:**

Creating a format for journal abstracts seems to be an effective intervention for presenting the message in articles.

## INTRODUCTION

It is well-documented that limited resources have increased the significance of knowledge transfer and attempts by the health sector’s decision makers in utilization of research results.^1^ Multiple studies have shown the gap between knowledge production and utilization of results. Such a gap can have more negative consequences in health care.^2^

Publication of articles seems to prepare favorable grounds for knowledge transfer, especially because academia is used to it and publications are considered as the criteria for their employment and promotion. According to previous studies, the most frequent method of knowledge transfer adopted by academics is publishing articles in scientific-research journals.^3^,^4^ Proper utilization of articles can therefore be considered an appropriate approach for improving the status of knowledge transfer. Article wording even affects its utilization. Michie and Johnston emphasize that correcting the wording in a way that specifies what, who, when, where and how a measure should be taken influences the implementation of knowledge. They believe that rewriting guidelines taking into account these tips is the most costeffective method.^5^

On the other hand the framework proposed for knowledge transfer by John Lavis et al^6^ and recommended by World Health Organization’s report on ‘Knowledge for Better Health’^7^ covers five steps: presence of a clear message (what), addressing of the direct target audience (who), the messenger (by whom), the method of transfer (how), and evaluating the effect of the message transferred (with what effect).^6^ Two of these five steps can be highlighted in each article: existence of a clear message and addressing of the direct target audience. By having a clear message we mean a clear mention of the research results in the abstract and context of the article upon which further actions can be taken. A group which receives the research results first-hand has been considered a direct target audience.^7^,^8^ In other words the frontline users of research results are direct target audiences.

Also, changing the format of journal articles e.g. formatted abstracts would raise the quality of the article.^9^ Some journals like BMJ have already started using formatted abstracts and ‘key messages box’ that highlights the salient findings of the study.^10^

This study questions to what extent researchers address their direct target audience in their articles, and whether a clear message has been written for these audiences. Until now, the authors have not come across a similar study in the data bases available.

## METHODS

### Study subjects

The articles published in maternal care, diabetes mellitus and tuberculosis programs were searched. These three themes were chosen because a national program exists in each, and also to cover a variety of diseases i.e. communicable diseases (tuberculosis; TB), non-communicable (diabetes mellitus), and to have a group at risk (pregnant women). Also, the maternal care and TB programs have worked on the millennium development goals.^11^

The articles published between 2001 and 2006 on an Iranian population in the abovementioned three topics were considered. A systematic search was done in international databases: Pubmed, and Embase; and domestic databases such as Iranmedex, SID (Scientific Information Database) and Iranpsych (Each one of these Iranian databases has been explained in additional file 2). Then, after reviewing the article titles and abstracts, short reports, case reports, basic science and irrelevant articles were excluded. Basic science articles are considered as articles whose direct target audiences are researchers, and have no direct application in service delivery and are solely done for scientific developments. The rest of the articles whose full texts were freely available via internet were included in the study and divided into clinical and health system research (HSR) studies. The study’s checklists were then completed. Clinical research was defined as a study whose direct target audience is clinical service providers (physicians and nurses etc). An HSR was considered as a study whose direct target audiences are policymakers, managers and/or experts not in direct contact with patients.

The journals of the selected articles were classified into three groups of clinical, HSR, and general (in case they covered both types of articles). The journals were grouped after examining four volumes of the concerned journals and their scope.

### Data gathering tools

The relevant data were collected through a checklist. The validity of the checklist was checked through literature review and expert opinions in a brain storming session. Twenty checklists were simultaneously completed by two individuals; a kappa of 0.8 represented a reliable inter rater agreement. The final version of the checklist was completed by two individuals independently. In case of a disagreement, a third person would intervene.

The variables of the study ‘examining the status of addressing the direct target audience, presence of a clear message in the article’ and their determinant factors included: type of study (clinical or HSR), journals publication site (domestic or international), abstract format (formatted or non-formatted), presence of a ‘key messages box’, and the corresponding author’s serving site (Ministry of Health and Medical Education [MOHME] universities or others). A ‘key messages box’ is a box containing the article’s message, what it has added to existing knowledge, and suggestions. Any kind of estimates (such as incidence or prevalence) and/or pointing towards the possibility of an association between two variables or in the form of an actionable message that specifies what should be done by whom and how is considered a message.^12^

### Statistical analysis

The descriptive statistics were calculated, and Logistic regression was used to analyze the association of determinant factors with ‘presence of a clear message’ and ‘addressing the direct target audience’ separately for the abstract and whole article (which includes the abstract too).

## RESULTS

### Characteristics of the articles under study

On the whole, 6167 articles (1060 articles on tuberculosis, 2631 on maternal care and 2476 articles on diabetes mellitus) were found between the years 2001 and 2006. Eventually, after excluding repetitive articles, case reports, short reports and basic scientific studies 1390 articles (235 on tuberculosis, 608 on maternal care and 547 on diabetes mellitus) remained which possessed the inclusion criteria. Out of 1390 articles only 795 articles full texts were accessible, so this number was studied.

**Figure 1:**
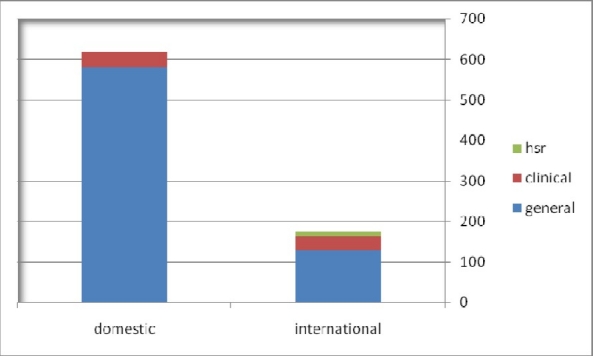
Distribution of included articles published in domestic and international journals

Among 795 articles, 564 articles were clinical (71%), and 231 (29%) were HSR studies. Six hundred and nineteen articles (77.9%) had been published in 68 domestic journals and the remainder had been published in 69 international journals. Ninety three percent of domestic articles had been published in general journals and 6% in clinical journals. Regarding international articles 73% had been published in general journals, 19% in clinical and 8% in HSR journals (figure 1).

An examination of the abstracts and whole articles yielded the following results:

*Abstract*: A clear message in the abstract was present in 95.7% of articles. And 3.1% of articles addressed the direct target audience in the abstract. Formatted abstracts were present in 70.6% of articles. Eight articles lacked an abstract section.

Table 1 demonstrates the effect of the factors associated with ‘presence of a clear message’ in the abstract. Logistic regression showed that ‘presence of a clear message’ was 3.59 times more in articles with a formatted abstract (CI 95%, 1.49-8.68) as compared to articles with non-formatted abstracts (p = 0.005). Likewise, an increase in the publication year of the article was associated with presence of a message in the abstract with an odds ratio of 0.75 (CI 95%, 0.57-1.00, p = 0.05). Meaning, with an increase in the publication year, the presence of a message in the abstract was reduced by 75%. Table 2 shows addressing of the direct target audience is twice as much in HSR article abstracts than in clinical ones. A higher number of articles published in domestic journals had a clear message and addressed the target audience in comparison to articles published in international journals. However, neither of these relationships were significant (Tables 1 and 2).

**Table 1:** Factors affecting presence of a clear message in the abstract

	Message								

	Present	Absent		Crude analysis			Logistic Regression	
	Number (%)	Number (%)	OR	Confidence interval	P	OR	Confidence interval	P
***Type of study***								
Clinical	541(96.8)	18(3.2)	1			1		
Health system	220(96.5)	8(3.5)	0.9	0.4-2.1	0.8	0.9	0.4-2.1	0.8
***Publication site***								
Domestic	595(97.2)	17(2.8)	1			1		
International	166(94.9)	9(5.1)	0.5	0.2-1.2	0.1	0.8	0.3-2.1	0.7
***Abstract format***								
Non-formatted	213(94.2)	13(5.8)	1			1		
Formatted	548(97.7)	13(2.3)	2.6	1.2-5.6	0.02	3.6	1.5-8.9	0.005
***Corresponding Author’s serving site***								
Other	9(90.0)	1(10.0)	1			1		
Ministry of Health and Medical Education (MOHME)	729(96.8)	24(3.2)	3.3	0.4-25	0.2	2.3	0.2-20.8	0.5
***Year of publication***								
2001	96(99)	1(10.0)	0.8	0.7-1.1	0.4	0.8	0.6-1.0	0.05
2002	112(95.7)	5(4.3)						
2003	102(98.1)	2(1.9)						
2004	143(97.3)	4(2.7)						
2005	152(96.8)	5(3.2)						
2006	156(94.5)	9(5.5)						

**Table 2:** Factors affecting addressing the direct target audience in the abstract

	Target audience								

	Present	Absent		Crude analysis			Logistic Regression	
	Number (%)	Number (%)	OR	Confidence interval	P	OR	Confidence interval	P
***Type of study***								
Clinical	14(2.5)	545(97.5)	1			1		
Health system	11(4.8)	217(95.2)	2	0.9-4.4	0.1	1.9	0.9-4.3	0.1
***Publication site***								
Domestic	23(3.8)	589(96.2)	1			1		
International	2(1.1)	173(98.9)	0.3	0.1-1.3	0.1	0.3	0.6-1.25	0.1
***Abstract format***								
Non-formatted	10(4.4)	216(95.6)	1			1		
Formatted	15(2.7)	546(97.3)	0.6	0.3-1.3	0.2	0.6	0.2-1.4	0.1
***Corresponding Author’s serving site***								
Other	0(0)	10(100)	1					
MOHME	24(3.2)	729(96.8)	1.0	1.02-1.04	1.8	*	-	*
***Year of publication***								
2001	2(2.1)	95(97.9)	1	0.8-1.2	0.7	1.1	0.9-1.5	
2002	6(5.1)	111(94.9)						
2003	2(1.9)	102(98.1)						
2004	6(4.1)	141(95.9)						
2005	5(3.2)	152(96.8)						
2006	4(2.4)	161(97.6)						

* The ‘Corresponding Author’s profession’ factor was zero, so it was omitted to prevent disrupting the table.

*Whole article (the abstract ‘and’ full text of the article)*: A clear message had been mentioned in at least one section of the article in 98.5% of articles, whereas, only 12.5% had addressed the direct target audience. Both ‘presence of a clear message’ and ‘addressing of the target audience’ in at least one section of the article were seen in only 12.2% of cases.

On studying the determinant factors of ‘presence of a clear message’ in the article through logistic regression, table 3 shows there was no significant relationship between any of these variables, though ‘presence of a clear message in at least one section of the article’ was seen 3.41 times more in articles with formatted abstracts (p = 0.06). An increase in the publication year affected ‘a clear message’ by an odds ratio of 0.85, without having a significant relationship with it (i.e. it reduced the presence of a clear message). Table 4 presents the factors affecting addressing of a direct target audience in at least one section of the article. The only variable that had a significant effect on addressing the direct target audience was ‘type of study’ (clinical or HSR), with an odds ratio of 2.3 (CI 95%, 1.47-3.48, p<0.001); this variable was seen almost twice as much in HSR than in clinical articles.

**Table 3:** Factors determining presence of a clear message in at least one section of the article

	Message								

	Present	Absent		Crude analysis			Logistic Regression	
	Number (%)	Number (%)	OR	Confidence interval	P	OR	Confidence interval	P
***Type of study***								
Clinical	555(98.4)	9(1.6)	1			1		
Health system	228(98.7)	3(1.3)	1.2	0.3-4.6	0.8	1.3	0.3-4.8	0.7
***Publication site***								
Domestic	611(98.7)	8(1.3)	1			1		
International	172(97.7)	4(2.3)	0.6	0.2-1.9	0.4	0.9	0.2-3.3	0.8
***Abstract form***								
Non-formatted	228(97.4)	6(2.6)	1					
Formatted	555(98.9)	6(1.1)	2.4	0.8-7.6	0.1	3.4	0.95-12.2	0.06
***Separate Conclusion***								
Absent	627(98.7)	8(1.3)	1			1		
Present	156(97.5)	4(2.5)	0.5	0.2-1.7	0.3	0.5	0.1-1.8	0.3
***Key messages box***								
Absent	781(98.5)	12(1.5)	1					
Present	2(100)	0(0)	1		0.9	*		*
***Corresponding Author’s serving site***								
Other	10(100)	0(0)	1					
MOHME	749(98.4)	12(1.6)	1		0.7	*		*
***Year of publication***								
2001	98(100)	0(0)	0.9	0.6-1.2	0.3	0.9	0.5-1.2	0.3
2002	116(97.5)	3(2.5)						
2003	105(98.1)	2(1.9)						
2004	147(100)	0(0)						
2005	156(98.1)	3(1.9)						
2006	161(97.6)	4(2.4)						

* The ‘Corresponding Author’s profession ’ and ’ knowledge transfer box’ factors were zero, so they were omitted to prevent disrupting the table

**Table 4:** Factors affecting addressing the direct target audience in at least one section of the article

	Target audience								

	Present	Absent		Crude analysis			Logistic Regression	
	Number (%)	Number (%)	OR	Confidence interval	P	OR	Confidence interval	P
***Type of study***								
Clinical	54(9.6)	510(90.4)	1			1		
Health system	45(19.5)	186(80.5)	2.3	1.5-3.5	0.000	2.3	1.5-3.5	0.000
***Publication site***								
Domestic	78(12.6)	541(87.4)	1			1		
International	21(11.9)	155(88.1)	0.9	0.6-1.6	0.8	0.9	0.5-1.5	0.6
***Abstract form***								
Non-formatted	35(15.0)	199(85.0)	1			1		
Formatted	64(11.4)	496(88.6)	0.7	0.5-1.1	0.2	0.7	1.6-0.4	0.2
***Separate Conclusion***								
Absent	76(12)	559(88.0)	1			1		
Present	23(14.4)	137(85.6)	1.2	0.8-2.0	0.4	1.3	0.8-2.2	0.3
***Key messages box***								
Absent	99(12.5)	694(87.5)	1					
Present	0(0)	2(100)	1.14	1.11-1.17	0.6	*		*
***Corresponding Author’s serving site***								
Other	0(0)	10(100)	1					
MOHME	95(12.5)	665(87.5)	1.14	1.11-1.17	0.2	*		*
***Year of publication***								
2001	15(15.3)	83(84.7)	1	0.9-1.1	0.5	1.0	0.9-1.2	0.7
2002	17(14.3)	102(85.7)						
2003	8(7.5)	99(92.5)						
2004	15(10.2)	132(89.8)						
2005	21(13.2)	138(86.8)						
2006	23(13.9)	142(86.1)						

* The ‘Corresponding Author’s profession’ and ‘knowledge transfer box’ factors were zero, so they were omitted to prevent disrupting the table

Similar to the abstract, tables 3 and 4 show that presence of a clear message and addressing of the direct target audience is higher in domestic articles, though this relationship was not significant.

## DISCUSSION

In the current study, only two out of 795 articles study had a ‘key messages box’. And 12.2% of articles had mentioned both a clear message and the target audience in at least one section of the article. Ninety five point seven percent of articles had a clear message in the abstract and 98.5% had it in at least one section of the article. The direct target audience had been addressed in the abstract in 3.1% of cases, and in at least one section of the article in 12.5%. Logistic regression analysis showed that addressing of the direct target audience was almost twice as much in HSR articles as compared to clinical ones (CI 95%, 1.49-3.51, p<0.001). Two reasons can explain this finding: firstly, HSR studies are done mostly on the basis of policy makers and managers needs, therefore emphasizing the target audience which is usually the granting body. The other reason may be the preassumption that the target audience and readers of clinical journals mainly consist of health service providers. Where the study was an HSR the target audience had been addressed, but where the above pre-assumption was present, the target audience had not been addressed. On the other hand, 19% of English articles and 6% of Persian articles had been published in clinical journals. This difference may be the reason a clear message and addressing of the target audience are more in domestic journals as compared to international ones.

Regarding selection bias it must be said that, in this study articles were chosen that were present in databases and whose full texts were accessible (No doubt lack of access to the full texts of articles was among the limitations of the study). There is a possibility that these journals were overestimated in our assessments, because articles whose journals are not registered in these databases and whose full texts are not accessible may be different.

Where information bias is concerned, the variables of ‘presence of a clear message’, ‘direct addressing of the direct target audience’, a questionnaire with a high kappa, and article reviewing by two independent persons (like systematic reviews), reduced the possibility of information bias.

The reason behind examining the abstracts and whole articles separately is that readers usually read either the abstract or the whole article, not the full text without its abstract. Therefore we took a practical approach in considering both sections.

Inadequacy of current knowledge transfer methods show the complicated process of converting ‘knowledge’ into ‘action’ requires multiple factors including a strong systematic framework, creativity, adequate skills and knowledge, recurrent followups and efforts at the organizational level, alongside the interaction between researcher and decision maker from the first to the final stages of research.^13^–^16^

The mode of writing, correct wording, writing a clear message and addressing the target audience are effective steps in knowledge transfer.^5^,^6^ Addressing the target audience and writing a clear message can be considered as correct wording too. Though it is better to evaluate the impact of mentioning the audience in using the message in future studies.

Albeit, it may be appropriate to say that knowledge utilization is more complex than simply delivering a message at the right time and to the right target audience. Not only do the target audience and message need to be identified, but it should also be known which question needs to be answered and which one has priority; how will the process of knowledge transfer be evaluated till it reaches ‘practice’. Based on publication of articles the final expected outcome is to promote the target audience’s knowledge.^17^,^18^

A clear message was present in 98.5% of articles. However, it was present 3.6 times more in articles with formatted abstracts in comparison to articles non-formatted abstracts (CI 95%, 1.5-8.9). Apparently, formatting abstracts improves the quality of the article.^9^ With this in mind, if journals outline a predefined format for abstracts it can have a significant effect on delivering a clear message in the abstract and consequently on knowledge transfer. Hence the results of this study can be a guide in the design of necessary interventions.

On the other hand, the findings of this research indicate that a formatted abstract does not have a significant effect on addressing the direct audience. Therefore considering a separate section with the heading ‘direct target audiences of the present study’ in journals especially general journals can be effective in the knowledge transfer process.

## CONCLUSIONS

Considering the fact that most articles did contain a clear message, and that it had a significant relationship with the abstract being formatted; and that the direct target audience had not been addressed in most cases, it seems that formatting articles and inserting a section named ’direct target audiences of the current study’ may be effective in the knowledge transfer process. However, a causal conclusion must be arrived at with care. Further studies may be necessary to confirm these conclusions.

## References

[1] Santesso N, Tugwell P (2006). Knowledge Translation in Developing Countries. J Contin Educ Health Prof.

[2] Lomas J, Enkin M, Anderson GM, Hannah WJ, Vayda E, Singer J (1991). Opinion leaders vs audit and feedback to implement practice guidelines. Delivery after previous cesarean section. JAMA.

[3] Nedjat S, Majdzadeh R, Gholami J, Nedjat S, Maleki K, Qorbani M (2008). Knowledge transfer in Tehran University of Medical Sciences: an academic example of a developing country. Implement Sci.

[4] Newton MS, Estabrooks CA, Norton P, Birdsell JM, Adewale AJ, Thornley R (2007). Health Researchers in Alberta: an explanatory comparison of defining characteristics and knowledge translation activities. Implement Sci.

[5] Michie S, Johnston M (2004). Changing clinical behaviour by making guidelines specific. BMJ.

[6] Lavis J N, Robertson D, Woodside J M, Mcleod C B, Abelson J (2003). How can research organizations more effectively transfer research knowledge to decision makers?. Milbank Q.

[7] (2004). World Report On Knowledge For Better Health: Strengthening Health Systems.

[8] Lomas L (1993). Diffusion, dissemination, and implementation: who should do what?. Ann N Y Acad Sci.

[9] Wong H, Truomg D, Mahamed A, Davidian C, Rana Z, Einarson T R (2005). Quality of structured abstracts of original research articles in the British Medical Journal, the Canadian Medical Association Journal and the Journal of American Medical Association: a 10-year follow-up study. Current Med Res Opin.

[10] (2009). 10) Author guidelines. BMJ.

[11] General U (2005). The Millennium Development Goals Report.

[12] Reardon R (2006). JLJGJ. From Research to Practice: A Knowledge Transfer Planning Guide.

[13] Kitson A, Ahmed LB, Harvey G, Seers K, Thomp-son DR (1996). From research to practice: One organizational model for promoting research-based practice. J Adv Nurs.

[14] Graham ID, Logan J, Harrison MB, Straus SE, Tetroe J, Caswell w (2006). Lost in knowledge translation: time for a map?. J Contin Educ Health Prof.

[15] Gagliardi A R, Fraser N, Wright F C, Lemieux-Charles L, Lemieux-Charles L (2008). Fostering knowledge exchange between researchers and decision-makers: Exploring the effectiveness of a mixed-methods approach.

[16] Dobbins M, Rosenbaum P, Plews N, Law M, Fysh A (2007). Information transfer: what do decision makers want and need from researchers?. Implement Sci.

[17] Almeida C, Bascolo E (2006). Use of research results in policy decision making, formulation, and implementation: A review of the literature. Cad Saude Publica, Rio de Janeiro.

[18] Lavis JN, Lomas L, Hamid H, Sewankambo N K (2006). Assessing country-level efforts to link research to action. Bull World Health Organ.

